# Cerebral fractional tissue oxygen extraction (cFTOE) during immediate fetal-to-neonatal transition: a systematic qualitative review of the literature

**DOI:** 10.1007/s00431-024-05631-2

**Published:** 2024-06-11

**Authors:** Christoph Schlatzer, Bernhard Schwaberger, Marlies Bruckner, Christina Helene Wolfsberger, Gerhard Pichler, Berndt Urlesberger, Nariae Baik-Schneditz

**Affiliations:** 1 Division of Neonatology, Department of Pediatrics and Adolescent Medicine, Medical University of Graz, Graz, Austria; 2 Research Unit for Neonatal Micro- and Macrocirculation, Department of Pediatrics and Adolescent Medicine, Medical University of Graz, Graz, Austria; 3 Research Unit for Cerebral Development and Oximetry Research, Medical University of Graz, Graz, Austria

**Keywords:** Cerebral fractional tissue oxygen extraction, cFTOE, FTOE, Near-infrared spectroscopy, Neonate, Transition

## Abstract

Cerebral monitoring during immediate fetal-to-neonatal transition is of increasing interest. The cerebral fractional tissue oxygen extraction (cFTOE) is a useful parameter to gain insight in the balance between tissue oxygen delivery and consumption during this complex process. The aim of this study was to review the literature on cFTOE during the first 15 min immediately after birth. A systematic qualitative literature research was last performed on 23 November 2023 of PubMed and EMBASE with the following search terms: neonate, infant, newborn, transition, after birth, delivery room, NIRS, near-infrared spectroscopy, spectroscopy, cFTOE, cerebral fractional tissue oxygenation extraction, cerebral oxygenation, and fractional oxygen extraction. Additional published reports were identified through a manual search of references in retrieved articles and in review articles. The methodological quality of the included studies was assessed by predefined quality criteria. Only human studies with data of cFTOE in the first 15 min after birth were included. Accordingly, exclusion criteria were defined as no measurement of cFTOE or no measurement within the first 15 min after birth. Across all studies, a total of 3566 infants (2423 term, 1143 preterm infants) were analysed. Twenty-five studies were identified describing cFTOE within the first 15 min after birth. Four studies established reference ranges for cFTOE and another four studies focused on the effect of pre-/perinatal circumstances on cFTOE in the first 15 min after birth. Six studies investigated the course of cFTOE after transition in infants without complications. Eleven studies analysed different potentially influencing parameters on cFTOE during transition.

* Conclusion*: This systematic review provides a comprehensive insight on cFTOE during uncomplicated transition as well as the influence of perinatal circumstances, respiratory, haemodynamic, neurological, and laboratory parameters in preterm and term infants.

**Table:** 

**What is Known:**	
• *The NIRS-measured cerebral fractional tissue oxygen extraction (cFTOE) is a useful parameter to estimate the balance between oxygen delivery and consumption*. • *During normal transition, the cFTOE decreases in the first minutes after birth and then remains at a stable plateau*.	
**What is New:**	
• *The cFTOE is a promising parameter that gives additional information on cerebral oxygenation and perfusion in preterm and term infants*. • *Several hemodynamic, metabolic, respiratory, and perinatal factors are identified, influencing the oxygen extraction of the newborn's brain after birth*.

• *The NIRS-measured cerebral fractional tissue oxygen extraction (cFTOE) is a useful parameter to estimate the balance between oxygen delivery and consumption*.

• *During normal transition, the cFTOE decreases in the first minutes after birth and then remains at a stable plateau*.

• *The cFTOE is a promising parameter that gives additional information on cerebral oxygenation and perfusion in preterm and term infants*.

• *Several hemodynamic, metabolic, respiratory, and perinatal factors are identified, influencing the oxygen extraction of the newborn's brain after birth*.

## Introduction

Recent guidelines recommend arterial oxygen saturation (SpO_2_) and heart rate (HR) monitoring during fetal-to-neonatal transition using pulse oximetry and optionally electrocardiogram (ECG) [1, 2]. Since these measurement methods do not provide any information about the brain perfusion and oxygenation, near-infrared spectroscopy (NIRS) became a non-invasive tool to monitor cerebral oxygenation during immediate transition. Cerebral regional oxygen saturation (crSO_2_) is the dominant parameter used and the number of NIRS studies is constantly increasing [3]. Oxygen delivery to the brain depends on the haemoglobin (Hb) concentration, its affinity to oxygen, and cerebral blood flow (CBF), which is affected by blood pressure and cerebrovascular resistance [4, 5]. In case of systemic hypoxia, two mechanisms are described to maintain oxygen availability to the brain. The first response is likely to be cerebral vasodilatation; if this response is ineffective or impaired, the relative cerebral oxygen extraction increases [6]. Increased oxygen extraction can be achieved by an increase in the surface for passive oxygen diffusion across capillaries, enabling tissues to extract more oxygen from the blood. In instances of decreased oxygen delivery, this mechanism can augment oxygen extraction from about 30% under normal circumstances to 50–60% [7]. A useful parameter to estimate the balance between oxygen delivery and consumption is the cerebral fractional oxygen extraction (cFOE) calculated by the following equation:

$$\mathrm{cFOE}=\mathrm{CMRO}2\;\left(\mathrm{cerebral}\;\mathrm{metabolic}\;\mathrm{rate}\;\mathrm{of}\;\mathrm{oxygen}\;\mathrm{consumption}\right)/\mathrm{COD}\;\left(\mathrm{cerebral}\;\mathrm{oxygen}\;\mathrm{delivery}\right)$$

Naulaers et al. showed in newborn piglets [8] that the NIRS-measured parameter cerebral fractional tissue oxygen extraction (cFTOE) calculated by the following equation:

$$\mathrm{cFTOE}=\left(\mathrm{SpO}2-\mathrm{crSO}2\right)/\mathrm{SpO}2$$

correlates well with FOE and can therefore be used as a proxy to measure FOE continuously and non-invasively. An increase in cFTOE can indicate either a decrease in oxygen delivery to the brain while the brain’s oxygen consumption remains constant or elevated, or it may suggest an increase in oxygen consumption not balanced by a corresponding rise in oxygen delivery. Conversely, a decrease in cFTOE suggests a reduction in the brain’s extraction of oxygen, which could result from decreased oxygen utilization or a steady level of oxygen consumption by the brain alongside an increased oxygen delivery [8]. 

The aim of this systematic qualitative review was to identify and summarize studies investigating on cFTOE in newborn infants during the first 15 min immediately after birth.

## Methods

Articles were identified using the stepwise approach specified in the Preferred Reporting Items for Systematic Reviews and Meta-Analysis (PRISMA) statement [9]. 

### Search strategy

The review research was performed through PubMed and EMBASE with the same search algorithm (Appendix) to identify English articles published from their inception to November 2023 with a predefined search algorithm. Search terms were “neonate, infant, newborn, transition, after birth, delivery room, NIRS, near-infrared spectroscopy, spectroscopy, cFTOE, cerebral fractional tissue oxygenation extraction, cerebral oxygenation and fractional oxygen extraction”. Additional published reports were identified through a manual search of references in retrieved articles and in reviews articles. Only human studies with NIRS monitoring in the first 15 min after birth were included.

### Study selection

**Fig. 1:**
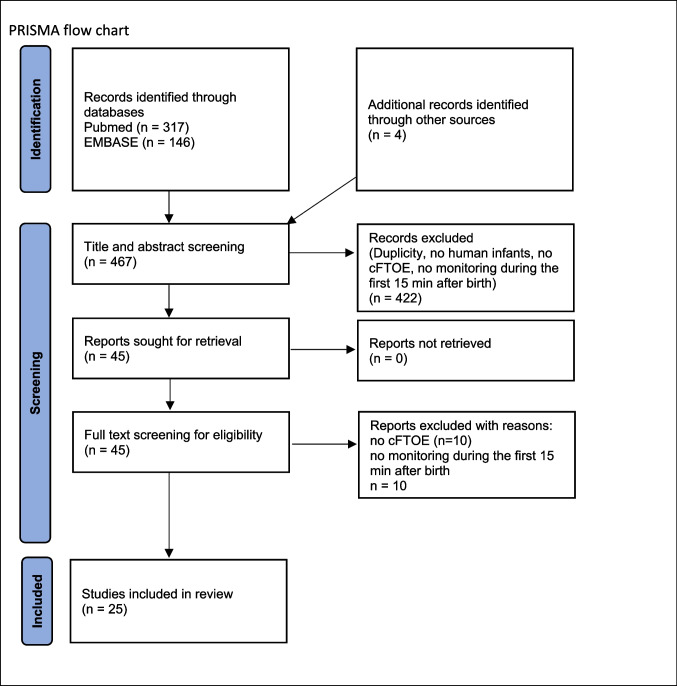
PRISMA flow chart

Two reviewers (C.S. and B.-S.N.) independently screened all of the study titles and abstracts for eligibility and critically appraised the full text of identified articles and assessed the methodological quality of included studies. Disagreements were resolved through discussion and consensus between the two authors (C.S. and B.-S.N.), who critically appraised the full text and assessed the methodological quality of the included studies. The primary outcomes were to review the literature on cFTOE in newborns in the first 15 min after birth and to stress its clinical relevance. All data were analysed qualitatively. One author (C.S.) did the data extraction by including the characterization of study type, patient demographics, methods, and results (Fig. 1).

### Assessment of methodological quality

**Fig. 2:**
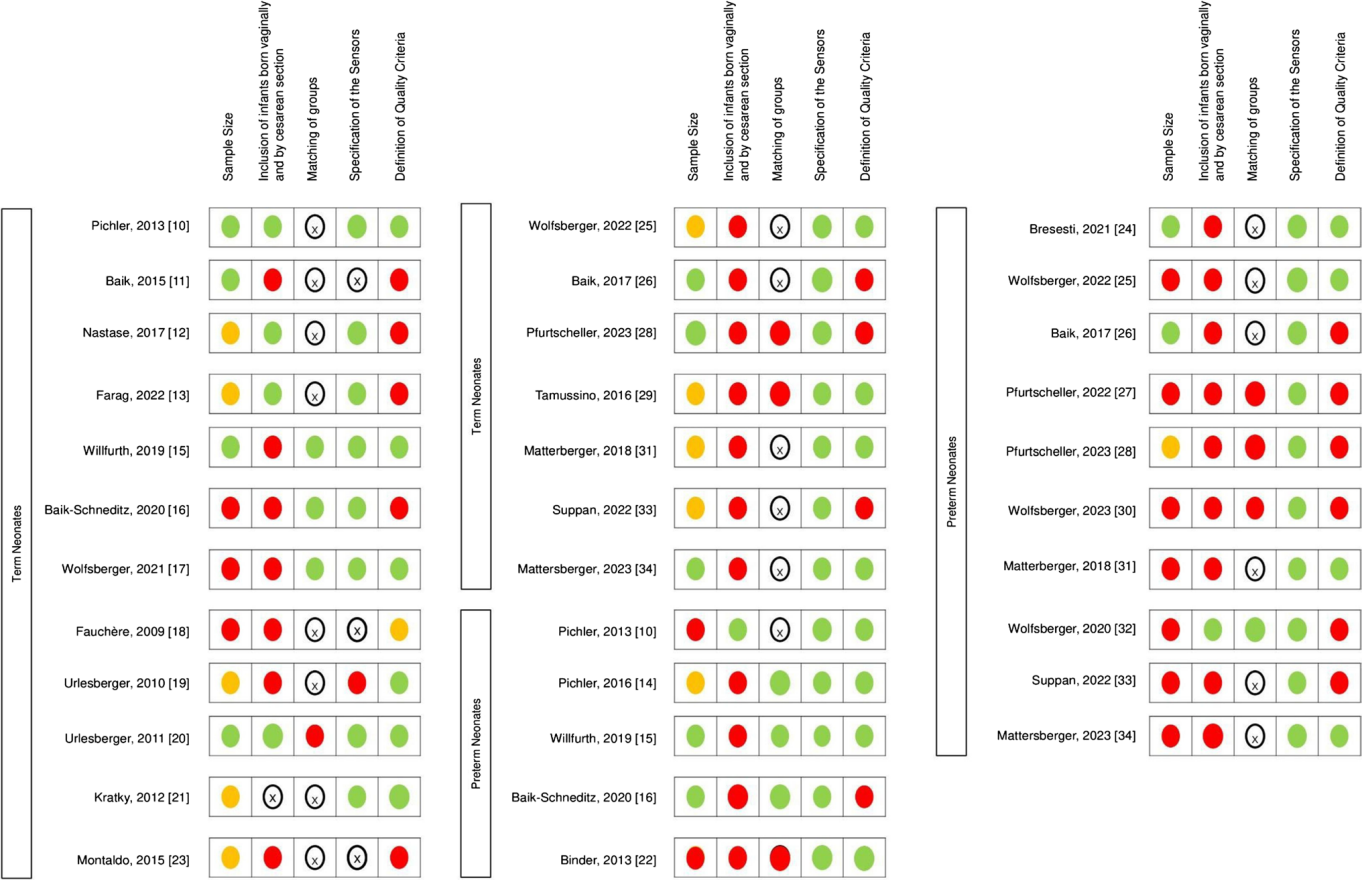
Methodological quality criteria: sample size (red, *n* ≤ 50; yellow, *n* = 50–100; green, *n* > 100); inclusion of infants born vaginally and by caesarean section (red, yes; X, not applicable; green, no); matching of investigated groups (red, yes; X, not applicable; green, no); specification of the used sensor (red, no; X, not applicable; green, yes); quality criteria to detect and eliminate artifacts defined (red, not defined; yellow, sparely defined; green, reported in detail)

The methodological quality of the included studies was assessed by the following criteria: (1) sample size, (2) inclusion of infants born vaginally and by caesarean, (3) matching of investigated groups, (4) specification of the used sensor, (5) definition of quality criteria to detect and eliminate artifacts (Fig. 2).

## Results

The initial research identified 317 articles through PubMed and 146 through EMBASE (Fig. 1). After removal of duplicates and rejection (i.e. no human studies, no monitoring in the first 15 min after birth, no cFTOE), 25 studies were included. All 25 studies performed crSO_2_ measurements during the first 15 min after birth and calculated cFTOE. Four studies established centiles for cFTOE in the first 15 min after birth [10–13]. Another four studies focused on the impact of pre-/perinatal circumstances on cFTOE [14–17]. Six studies investigated the course of cFTOE within the first 15 min after birth [18–23]. Eleven studies analysed different potentially influencing factors (i.e. respiratory [24, 25] and haemodynamic [26–28], neurological [29, 30], and laboratory parameters [31–34]) on cFTOE during neonatal transition (Table 1).

**Table 1:** Characteristics of the included studies, listed according to the headings of the discussion and year of publication

Author/year	Study design		Gestation	Neonates (*n*)	Mode of delivery	Respiratory support	Device	Position	Duration	PMID
cFTOE and centiles										
Pichler, 2013 [10]	Prospective observational study		Term and preterm	381	CS/VD	No	INVOS 5100	Frontal left	15 min	23972642
Baik, 2015 [11]	Prospective observational study		Term	140	CS	No	NIRO 200NX	Frontal	15 min	26330229
Nastase, 2017 [12]	Prospective observational study		Term	74	CS/VD	No	INVOS 5100 C	Frontal left	10 min	-
Farag, 2022 [13]	Prospective obervational study		Term	60	CS/VD	No	INVOS 5100 C	Frontal left	10 min	-
cFTOE and prenatal (intrauterine growth restriction [16], tobacco exposure [17])/perinatal circumstances (cord clamping time [14], maternal anesthesia [15])										
Pichler, 2016 [14]	Post hoc analysis of observational studies		Preterm	72	CS	Yes	INVOS 5100 C	Frontal left	15 min	26103783
Willfurth, 2019 [15]	Post hoc analysis of observational studies		Term and preterm	733	CS	Yes/no	INVOS 5100	Frontal left	15 min	31096224
Baik-Schneditz, 2020 [16]	Retrospective observational cohort study		Term and preterm	180	CS	Yes/no	INVOS 5100 C	Frontal left	15 min	32516786
Wolfsberger, 2021 [17]	Post hoc analysis of observational study		Term	24	CS	No	INVOS 5100 C	Frontal left	15 min	34888265
Course of cFTOE in the first 15 min after birth										
Fauchère, 2009 [18]	Observational study		Term	17	CS	No	NIRO 300	Scalp	15 min	19914638
Urlesberger, 2010 [19]	Prospective observational study		Term	61	CS	No	INVOS 5100	Frontal left	15 min	20955848
Urlesberger, 2011 [20]	Prospective observational study		Term	107	CS/VD	No	INVOS 5100 C	Frontal left	15 min	21481417
Kratky, 2012 [21]	Prospective observational study		Term	63	VD	No	INVOS	Frontal left	15 min	22173332
Binder, 2013 [22]	Prospective observational study		Preterm	49	CS	Yes/no	INVOS	Frontal left	15 min	23434123
Montaldo, 2015 [23]	Prospective observational study		Term	61	CS	No	EQUANOX 7600	Frontal	15 min	25933924
cFTOE and respiratory parameters (hypoxemia [24], carbon dioxide [25])										
Bresesti, 2021 [24]	Retrospective analysis of four studies		Preterm	150	CS	Yes	INVOS 5100 C/NIRO 200NX	Frontal left	15 min	34048860
Wolfsberger, 2022 [25]	Post hoc analysis of observational studies		Term and preterm	95	CS	No	INVOS 5100 C	Frontal left	15 min	34710875
cFTOE and haemodynamic parameters (blood pressure [26, 27], cardiac output [28])										
Baik, 2017 [26]	Observational study		Term and preterm	478	CS	Yes/no	INVOS 5100 C	Frontal left	15 min	28427056
Pfurtscheller, 2022 [27]	Post hoc analysis of observational studies		Preterm	47	CS	Yes/no	INVOS 5100 C	Frontal left	15 min	36210957
Pfurtscheller, 2023 [28]	Post hoc analysis of observational studies		Term and preterm	286	CS	Yes/no	INVOS 5100 C	Frontal left	15 min	36880893
cFTOE and neurological parameters (brain activity [29], neurodevelopment [30])										
Tamussino, 2016 [29]	Prospective observational study		Term	59	CS	Yes/no	INVOS	Frontal left	15 min	27039154
Wolfsberger, 2023 [30]	Retrospective analysis of observational studies		Preterm	42	CS	Yes	INVOS 5100 C	Frontal left	15 min	36997902
cFTOE and laboratory parameters (blood glucose [31], interleukin-6 [32], fetal haemoglobin [33], acid-base status [34])										
Matterberger, 2018 [31]	Post hoc analysis of observational studies		Term and preterm	75	CS	Yes/no	INVOS 5100	Frontal left	15 min	29958674
Wolfsberger, 2020 [32]	Post hoc analysis of observational studies		Preterm	46	CS/VD	Yes/no	INVOS 5100 C	Frontal left	15 min	32793528
Suppan, 2022 [33]	Prospective observational study		Term and preterm	109	CS	No	INVOS 5100 C	Frontal left	15 min	35882188
Mattersberger, 2023 [34]	Post hoc analysis of observational studies		Term and preterm	157	CS	Yes/no	INVOS 5100	Frontal left	15 min	37196035

*CS *caesarean section, *VD* vaginally delivered

## Discussion

This is the first systematic review focusing on cFTOE during the first 15 min after birth in term and preterm infants.

### cFTOE and centiles

Four studies have established centiles for cFTOE in the initial minutes immediately after birth, using two distinct devices: INVOS 5100 C (Somanetics, Troy, Michigan), utilized by Pichler et al. [10], Nastase et al. [12], and Farag et al. [13]; and the NIRO 200NX device (Hamamatsu, Japan), used by Baik et al. [11] Three of these studies exclusively focused on term infants, while Pichler et al. also included preterm infants (gestational age [GA] in weeks; mean ± SD, GA 34.9 ± 1.4 weeks). All studies assessed infants delivered by caesarean section and except for Baik et al., additionally encompassed vaginally delivered. Each study exclusively examined infants without medical support.

Farag et al. [13] and Nastase et al. [12] conducted measurements at minutes 1, 5, and 10 after birth, while Pichler et al. [10] and Baik et al. [11] initiated measurements from minute 2 continuously describing data at each minute until minute 15 after birth. Across all four studies, a consistent pattern emerged: cFTOE decreased in the initial 5 min after birth and subsequently maintained relatively constant.

Comparing the 50th percentiles of different studies, Farag et al. and Nastase et al. reported similar values in minute 1 after birth. When looking at the corresponding 50th percentile of the crSO_2_ values in these two studies, Farag et al. showed higher crSO_2_ and SpO_2_ values, which might be attributed to the routinely done maternal oxygen administration through a mask with a fractional inspired oxygen (FiO_2_) of 0.4 in cases of caesarean section. Despite this divergence in crSO_2_, the cFTOE was similar, which suggests that the oxygen consumption remains stable in the first minute after birth. In minute 5, where the other two studies also reported values, the 50th percentile values across all four studies exhibited good correlation. However, by minute 10, Nastase et al. and Baik et al. demonstrated higher 50th percentile values compared to the other two studies, introducing a slight divergence in results. When SpO_2_ rises, the cTOI of the NIRO 200NX displays higher values than the crSO_2_ measured by the INVOS oximeter [11], which might explain the higher 50th percentile of Baik et al.’s study in minutes 10 and 15. Farag et al., Nastase et al., and Pichler et al. utilized the INVOS 5100 device, with Pichler et al. introducing the highest sample size and thus, the highest validity in results. Results from Farag et al. closely aligned with those of Pichler et al., while Nastase et al.’s study exhibited some variance, potentially attributed to its smaller sample size (Table 2).

**Table 2:** Descriptive analysis of the 10th, 50th, and 90th percentile of established cFTOE centiles

Time point		1 min			5 min			10 min			15 min			T/PT	Mode of delivery	
Centiles		10th	50th	90th	10th	50th	90th	10th	50th	90th	10th	50th	90th			
Device	INVOS 5100 C	0.24	0.44	0.67	0.08	0.22	0.32	0.11	0.21	0.27				T	CS/VD	Nastase, 2017 [12]
	INVOS 5100 C	0.19	0.45	0.73	0.11	0.18	0.35	0.08	0.13	0.26				T	CS/VD	Farag, 2022 [13]
	INVOS 5100				0.06	0.21	0.45	0.05	0.15	0.31	0.07	0.18	0.34	T + PT	CS/VD	Pichler, 2013 [10]
	NIRO 200NX				0.1	0.2	0.35	0.09	0.21	0.35	0.13	0.24	0.37	T	CS	Baik, 2015 [11]

*T *term, *PT *preterm, *CS *caesarean section, *VD *vaginally delivered

### cFTOE and pre-/perinatal circumstances

Wolfsberger et al. conducted a study revealing that term infants exposed to prenatal tobacco showed a significantly higher cFTOE as well as a significantly lower crSO_2_ within the initial 5 min after birth. These findings suggest an elevated risk of cerebral hypoxia in infants with prenatal tobacco exposure immediately following delivery [17]. Additionally, infants experiencing intrauterine growth restriction demonstrated a decrease in cFTOE slightly above the 10% threshold in minutes 11 to 13 after birth. This reduction was attributed to a combination of increased cerebral oxygen delivery and diminished cerebral oxygen consumption, indicative of the fetus’ adaptive response to chronic intrauterine hypoxia [16]. 

Two studies focused on perinatal circumstances. In the first study, Willlfurth et al. [15] examined the impact of maternal anesthesia on cerebral oxygenation. Despite significant differences in SpO_2_, HR, and provided FiO_2_, no significant difference in cFTOE was observed between general and spinal anesthesia. Similar cFTOE values in term infants were proposed to be a result of cerebral autoregulatory mechanisms maintaining cerebral oxygen delivery and/or reduced cerebral oxygen consumption following maternal general anesthesia. In preterm infants, higher FiO_2_ levels used during transition were considered responsible for comparable values, despite compromised breathing efforts in the general anesthesia group [15].

The second study investigated the effects of delayed cord clamping (DCC) in preterm infants [14]. The study demonstrated that DCC led to higher cFTOE compared to early cord clamping (ECC), aligning with the trend of lower SpO_2_ in the third minute after birth, along with reduced HR and Apgar scores in the DCC group during the initial minutes after cord clamping. An explanation for this observation may be the increase in carotid arterial pressure and flow resulting from the loss of low-pressure placental circulation, causing an immediate rise in afterload in the ECC group [14]. 

### Course of cFTOE and respiratory/haemodynamic parameters

Two studies showed that the cFTOE decreases in the first minutes after birth and then remains at a stable plateau in term infants [19, 21], along with the findings of aforementioned centile studies [10–13]. It was speculated that this decline could be a response to the increase in oxygen delivery after birth [19, 21]. Conversely, Fauchère et al. observed a consistent cFTOE over time, suggesting constant oxygen consumption. A possible reason is the lower number of patients in their study. Further, the cFTOE values in their study were lower in the first 4 min which could be explained by the accordingly lower median SpO_2_ values in these minutes and thus lower oxygen delivery [18]. 

Another study by Binder et al. divided preterm infants into two groups according to if they received respiratory support or not, finding that those with support exhibited a less pronounced decrease in cFTOE. This was hypothesized to be a compensatory mechanism wherein the brain extracts more oxygen due to reduced tissue oxygen delivery [22]. The reduced oxygen delivery might be explained by the study of Pfurtscheller et al. [28]. In their study, involving also preterm infants with and without respiratory support, emerged a significant correlation between higher cardiac output (CO) and lower cFTOE values. Notably, the gestational age of infants with respiratory support was lower compared to preterm infants without respiratory support. The authors suggested that this correlation reflected passive pressure-dependent cerebral perfusion, providing evidence of impaired cerebral autoregulation in this particular group of compromised preterm infants receiving respiratory support [28].

Since the blood pressure amplitude depends on CO, two studies underscore the results by Pfurtscheller et al. by examining the correlation between blood pressure and cFTOE. The first study explored the potential impact of mean arterial blood pressure (MABP) in minute 15 after birth on crSO_2_ and cFTOE in preterm and term infants with and without respiratory support [26]. Their findings revealed a significant negative correlation between cFTOE and MABP in preterm infants, indicating that cFTOE increased with decreasing MABP. In the same study, preterm infants showed significantly higher cFTOE values compared to term infants. The authors concluded that the revealed blood-pressure-dependent cerebral perfusion in preterm infants might possibly be attributed to a compromised cerebral autoregulation [26]. 

The second study delved further into this topic by differentiating between preterm infants with and without respiratory support [27]. The study identified a significant negative correlation between systolic, diastolic, and mean arterial blood pressure and cFTOE in preterm infants with respiratory support, whereas in infants without respiratory support no correlations were observed. This finding reinforced the notion of passive pressure-dependent cerebral perfusion, indicating impaired cerebral autoregulation especially in compromised preterm infants with respiratory support. Further, the respiratory support itself might also have an influence. The higher intrathoracic pressure through the mask ventilation may lead to lower venous blood return to the heart and therefore lower stroke volume, which may result in compromised brain oxygenation parameters [27]. 

In a comparative study involving cFTOE, renal fractional tissue oxygenation extraction (rFTOE), and mesenteric fractional tissue oxygen extraction (mFTOE), it was found that rFTOE and mFTOE remained elevated in the first 6 min after birth with a following decrease reaching a stable plateau in minute 10, whereas the cFTOE decreased in the first minutes reaching a stable plateau at minute 7. This was attributed to a transient persistence of right-to-left ductal and/or atrial shunts, as evidenced by significant differences in pre- and postductal SpO_2_ measurements [23]. Alternatively, Urlesberger et al. retrieved in their study also peripheral regional oxygen saturation (prSO_2_) in the tissue of the right forearm and left calf with near-infrared spectroscopy in term infants after caesarean section and showed that the prSO_2_ was lower than crSO_2_ in the first minutes after birth and suggested centralization, leading to reduced oxygen delivery and vasoconstriction in non-vital organs [19]. 

The combined effect of hypoxemia and bradycardia on cFTOE was explored in another study, revealing that this combination led to higher cFTOE compared to bradycardia alone. This implies that cerebral tissue is less challenged by bradycardia alone, highlighting the importance of promptly titrating FiO_2_ to ensure adequate oxygen delivery in the early minutes after birth [24]. 

Examining the influence of pCO_2_ on cFTOE in preterm and term infants after caesarean section, Wolfsberger et al. identified a positive correlation in preterm infants. Several explanations were listed: (i) infants with higher pCO_2_ levels showed a trend to lower SpO_2_ and pO_2_, resulting in reduced oxygen delivery to the brain and increased oxygen extraction; (ii) the vasodilatory effect of pCO_2_ might not outweigh the vasoconstrictive effect of rising pO_2_ levels in preterm infants; (iii) preterm infants showed higher fetal haemoglobin (HbF) values than term infants and the consequential higher affinity to haemoglobin might counteract the right shift of the oxygen dissociation curve (ODC) due to high pCO_2_ levels; and (iv) the less pronounced closure of the ductus arteriosus in preterm infants might be associated with a higher steal phenomenon resulting in a decrease of CBF and oxygen delivery to the brain [25]. 

 Concerning mode of delivery, Urlesberger et al. found except for 1 minute (in minute 10, infants delivered by caesarean section had a lower cFTOE) no significant differences in cFTOE course in the first 10 min after birth in vaginally and via caesarean section delivered term infants [20], concordant with the results of Pichler et al. [10]. In contrast, Farag et al. and Nastase et al. found significant higher cFTOE in infants born via caesarean section [12, 13]. This finding was attributed to higher brain oxygenation metrics and SpO_2_ in vaginally delivered infants which was explained by firstly, higher pCO_2_ levels which led to cerebral vasodilatation and consequential more oxygen delivery. Secondly, lung fluid is more rapidly cleared in vaginally delivered infants. Thirdly, vaginally delivered infants have higher catecholamine levels, which constrict peripheral blood vessels and thus more CBF [13]. The higher CBF is underscored by the findings of Morimoto et al. [35], which showed that the cerebral blood volume (CBV) is higher in vaginally delivered infants in the first 4 min after birth. It was concluded that this difference in CBV is a response to hypoxia during the passage through the birth canal, which causes parasympathetic and sympathetic outflow and peripheral vasoconstriction with consequential increasing CBF [35].

In summary, these studies collectively shed light on various factors influencing cFTOE in the early minutes after birth, encompassing oxygen delivery and respiratory support. The nuanced findings underscore the complexity of cerebral oxygen dynamics and their potential implications for neonatal health.

### cFTOE and brain activity/neurodevelopment

Tamussino et al. classified term infants based on amplitude-integrated electroencephalogram (aEEG) voltage levels during the first 10 min after birth (study group, low voltage with a minimal (Vmin) < 5 µV or maximal amplitude (Vmax) < 10 µV; control group, normal voltage aEEG and no respiratory support). The study group with low voltage exhibited significantly higher cFTOE, attributed to primarily lower oxygen delivery with depletion of oxygen to maintain low activity in the study group [29]. 

Regarding neurodevelopmental outcome, Wolfsberger et al. stratified preterm infants according to their long-term outcome at a corrected gestational age of 2 years and found in the adverse outcome group besides a lower gestational age a higher cFTOE in almost all (11 out of 14) first minutes after birth, while there were no significant differences in the routine monitoring parameters SpO_2_, HR and, except for 1 min, in the provided FiO_2_. It was suggested that a lower crSO_2_ and consecutive higher cFTOE in the first minutes after birth have an impact on the neurodevelopmental long-term outcome [30]. 

### cFTOE and laboratory parameters

Acid-base status and metabolic parameters are crucial indicators of sufficient oxygenation during the immediate transition, providing valuable insights for predicting outcomes and guiding interventions. In their study, Matterberger et al. [31] identified a significant positive correlation between low blood glucose (within the normal ranges) and cFTOE in term and preterm infants. This association was attributed to low blood glucose inducing vasodilatation, subsequently increasing cerebral blood flow and oxygen supply.

Furthermore, this association was more pronounced in preterm infants when compared to term infants. The heightened correlation between blood glucose and cFTOE in preterm infants suggests potential impairments in cerebral autoregulatory mechanisms compared to term infants. In another study by Mattersberger et al. [34], cFTOE in preterm infants showed a positive correlation with lactate, a negative correlation with pH-value and base excess, and no correlation with bicarbonate. In term infants, a significantly positive correlation was solely found between cFTOE and bicarbonate.

The explanation of these findings might be that elevated lactate levels result in pulmonary vasoconstriction and low pH values in reduced contractility of cardiomyocytes and reduced responsiveness to catecholamines. Both mechanisms may contribute to impaired CO in preterm infants, leading to reduced oxygen delivery to the brain, heightened oxygen consumption, and an increased cFTOE. Hence, it was hypothesized that as gestational age advances, the dependency of cerebral autoregulation mechanisms on acid-base status diminishes, resulting in a more stable maintenance of cerebral oxygen supply in term infants [34]. 

Two studies analysed the impact of laboratory findings on cFTOE. Wolfsberger et al. stratified preterm infants based on their interleukin-6 values from umbilical cord blood, creating a group with fetal inflammatory response syndrome (FIRS) and a group without FIRS. In the FIRS group, cFTOE values were significantly lower in the first 4 min after birth, suggesting compromised oxygen consumption and delivery during this critical period. The authors proposed that, since cFTOE predominantly reflects the venous compartment of the blood, FIRS-induced centralization and potential alterations in the ratio of arterial, capillary, and venous compartments could impact cFTOE [32]. 

Suppan et al. conducted a study investigating the impact of fetal haemoglobin (HbF) on cFTOE. The results revealed a significant negative correlation in preterm infants, indicating that higher levels of HbF were associated with lower cFTOE within the first 5 min after birth. The study proposed several explanatory factors: (i) shift of the ODC: In preterm infants, elevated HbF levels may induce a leftward shift in the oxygen dissociation curve. This shift could influence oxygen parameters, affecting the release of oxygen to tissues. (ii) Differences in metabolic rates: Preterm infants might exhibit distinct metabolic rates, which could contribute to differences in oxygen consumption. (iii) Compensation mechanism: The authors suggested that the delivery of oxygen to the brain could be modulated by changes in cerebral blood flow to counterbalance disparities in haemoglobin affinity. However, in preterm infants, this compensatory mechanism might be compromised, resulting in reduced oxygen extraction at higher levels of HbF [33]. These findings illuminate intricate interactions between HbF levels and oxygen dynamics during the critical early minutes after birth in preterm infants.

## Conclusion

In conclusion, cFTOE is a promising value that gives additional information on cerebral oxygenation and perfusion in preterm and term infants during neonatal transition. The findings highlight the intricate interplay between haemodynamic, metabolic, respiratory, and perinatal factors in shaping cerebral oxygenation in the crucial period after birth. The identification of centiles and associations with long-term outcomes contributes valuable insights into neonatal physiology and the potential impact for clinical care in future. Further research and exploration of these parameters are warranted to deepen our understanding and refine clinical practices in neonatal care.

## Data Availability

The original contributions presented in this study are included in the article/supplementary material; further inquiries can be directed to the corresponding author.

## Appendix

Search strategies used for the systematic review

#1 newborn OR infant OR neonate

#2 near-infrared spectroscopy OR NIRS OR spectroscopy

#3 transition OR after birth OR delivery room

#4 cFTOE OR cerebral fractional tissue oxygenation extraction OR cerebral oxygenation OR fractional oxygen extraction

Search strategy: #1 AND #2 AND #3 AND #4

Search strategy for PubMed and EMBASE: last performed on 23/11/2023

## Abbreviations

aEEG: Amplitude-integrated electroencephalogram
CBF: Cerebral blood flow
CBV: Cerebral blood volume
cFOE: Cerebral fractional oxygen extraction
cFTOE: Cerebral fractional tissue oxygen extraction
CMRO_2_: Cerebral metabolic rate of oxygen consumption
CO: Cardiac output
COD: Cerebral oxygen delivery
crSO_2_: Cerebral regional oxygen saturation
CS: Caesarean section
DCC: Delayed cord clamping
ECC: Early cord clamping
ECG: Electrocardiogram
FiO_2_: Fractional inspired oxygen
FIRS: Fetal inflammatory response syndrome
GA: Gestational age
Hb: Haemoglobin
HbF: Fetal haemoglobin
HR: Heart rate
MABP: Mean arterial blood pressure
mFTOE: Mesenteric fractional tissue oxygen extraction
NIRS: Near-infrared spectroscopy
ODC: Oxygen dissociation curve
PRISMA: Preferred Reporting Items for Systematic Reviews and Meta-Analysis
prSO_2_: Peripheral regional oxygen extraction
PT: Preterm
rFTOE: Renal fractional tissue oxygen extraction
SpO_2_: Arterial oxygen saturation
T: Term
VD: Vaginally delivered
